# Distinct Roles for Bruton's Tyrosine Kinase in B Cell Immune Synapse Formation

**DOI:** 10.3389/fimmu.2018.02027

**Published:** 2018-09-06

**Authors:** Sara Roman-Garcia, Sara V. Merino-Cortes, Sofia R. Gardeta, Marjolein J. W. de Bruijn, Rudi W. Hendriks, Yolanda R. Carrasco

**Affiliations:** ^1^B cell Dynamics Laboratory, Department on Immunology and Oncology, Centro Nacional de Biotecnología (CNB)-CSIC, Madrid, Spain; ^2^Department of Pulmonary Medicine, Erasmus University Medical Center, Rotterdam, Netherlands

**Keywords:** B cells, Btk, immune synapse, actin cytoskeleton, shuttling/scaffold, kinase, cell activation

## Abstract

Bruton's tyrosine kinase (Btk) has a key role in the signaling pathways of receptors essential for the B lymphocyte response. Given its implication in B cell-related immunodeficiencies, leukemias/lymphomas and autoimmunity, Btk is studied intensely and is a target for therapy. Here, using primary B cells from distinct mouse models and the pharmacological inhibitors ibrutinib and acalabrutinib, we report distinct roles for Btk in antigen-triggered immune synapse (IS) formation. Btk recruitment to the plasma membrane regulates the B cell ability to trigger IS formation as well as its appropriate molecular assembly; Btk shuttling/scaffold activities seem more relevant than the kinase function on that. Btk-kinase activity controls antigen accumulation at the IS through the PLCγ2/Ca^2+^ axis. Impaired Btk membrane-recruitment or kinase function likewise alters antigen-triggered microtubule-organizing center (MTOC) polarization to the IS, B cell activation and proliferation. Data also show that, for B cell function, IS architecture is as important as the quantity of antigen that accumulates at the synapse.

## Introduction

Bruton's tyrosine kinase (Btk) belongs to the Tec family of non-receptor tyrosine kinases. Btk has a central role in BCR signaling, but also participates in that of chemokine, Toll-like and Fc receptors (1). Btk mutations cause immunodeficiencies in humans (XLA, X-linked agammaglobulinemia) and mice (Xid). Btk-kinase inhibitors have anti-tumor activity in patients with B cell malignancies and also reduce autoimmune symptoms (1, 2). Structurally, Btk has an N-terminal pleckstrin homology (PH) domain able to bind phospholipids, a Tec homology (TH) domain, SH3 and SH2 domains, and a C-terminal catalytic domain (1). For activation downstream of the BCR, Btk is recruited from cytosol to the plasma membrane by phosphatidylinositol (3,4,5)-trisphosphate (PIP_3_) through the PH domain, followed by phosphorylation at residue Y551 by Lyn/Syk, and auto- or transphosphorylation at Y223 (3). Then, Btk binds to phosphorylated SLP65 and activates phospholipase Cγ2 (PLCγ2), which triggers Ca^2+^ mobilization and diacylglycerol (DAG)-dependent pathways (3). Btk also has scaffold/shuttling functions; it brings phosphatidylinositol-4-phosphate 5-kinase (PIP5KI) from the cytosol to the plasma membrane to produce phosphatidylinositol (4,5)-bisphosphate (PIP_2_), a substrate of PLCγ2, but also of PI3K to generate PIP_3_ (4). Btk is implicated in integrin activation downstream of the BCR (5), and in actin dynamics to promote BCR microcluster assembly, cell spreading and antigen processing (6–8).

BCR recognition of antigen triggers the establishment of a platform for B cell/antigen-presenting cell (APC) interaction termed the immune synapse (IS). The IS is stabilized by assembly of an integrin- and F-actin-rich peripheral domain (peripheral supramolecular activation cluster, pSMAC) that delimits a central territory characterized by BCR/antigen accumulation (central SMAC, cSMAC) (9–12). The tyrosine kinases Lyn and Syk, as well as PI3K, Vav proteins, Rac and Rap GTPases, and the Wiskott-Aldrich syndrome protein (WASP) participate in cytoskeletal rearrangement and in LFA-1 integrin activation, both downstream of the BCR (6, 13–16). Syk promotes recruitment of the scaffold protein vinculin to the pSMAC, where it strengthens B cell adhesion by linking the integrin clustering with the cortical actin cytoskeleton (12, 17, 18). Vinculin recruitment to the IS follows an early wave of PIP_2_ production by the PIP5KI (12); PIP_2_ is needed for vinculin activation and translocation to the plasma membrane (19). The IS provides a framework for signaling and polarized membrane trafficking to achieve B cell activation and antigen extraction from the APC (20).

Here we studied the role of Btk- shuttling/scaffold and -kinase activities in IS formation of mouse primary B cells. We found that Btk controls the B cell ability to trigger IS formation and the assembly of the pSMAC mainly through the shuttling/scaffold activities; regulation of PIP_2_ production at the plasma membrane seems to be involved. Btk kinase activity determines antigen accumulation at the cSMAC by controlling the PLCγ2/Ca^2+^ axis. Btk-deficient B cells nonetheless showed no alterations in IS formation, suggesting dominant negative effects for Btk when is functionally altered. Defects in Btk membrane recruitment or kinase activity affects antigen-triggered microtubule-organizing center (MTOC) polarization to the IS, B cell activation and proliferation. Our data also indicated that appropriate IS molecular assembly is as important as the quantity of antigen accumulated at the synapse for B cell function.

## Materials and methods

### Mice and B cell isolation

Primary B lymphocytes were isolated from spleens of adult (8- to 20-week-old) wild type and Btk-KO mice (21) (both in C57BL/6J genetic background), and of wild type CBA/Ca mice and Xid (CBA/N) (22), by negative selection using mouse pan-T Dynabeads (DynaI Biotech, Invitrogen) in the total splenocytes obtained after a Lympholyte step (Cedarlane Laboratories); we enriched to >90% B cells. Animal procedures were approved by the CNB-CSIC Bioethics Committee and conform to institutional, national and EU regulations. B cells were cultured in complete RPMI (10 mM Hepes, 2 mM L-Glu and 50 μM β-mercaptoethanol) supplemented with 10% FCS.

### Real-time microscopy on planar lipid bilayers

Artificial planar lipid bilayers were assembled in FCS2 chambers (Bioptechs) as described (11). Briefly, unlabeled murine GPI-linked ICAM-1-containing 1,2-dioleoyl-PC (DOPC) liposomes and DOPC liposomes containing biotinylated lipids were mixed with DOPC liposomes at distinct ratios to obtain specified molecular densities (ICAM-1 at 200 molec/μm^2^; biotin-lipids, as indicated). Artificial planar lipid bilayers were assembled on sulphochromic solution-treated coverslips in FCS2 closed flow-chambers (Bioptechs), and blocked with PBS/2% FCS (1 h, room temperature (RT)). Surrogate antigen was tethered to membranes by incubation with Alexa Fluor 647 or 555 streptavidin (Molecular Probes), followed by monobiotinylated rat anti-κ light chain monoclonal antibody (mAb; clone187.1). Monobiotinylation was achieved labeling the antibody with 1 μg/ml NHS-LC-LC-Biotin (30 min, RT, in PBS; Pierce), followed by dialysis and checked by FACS. We estimated the number of molecules/μm^2^ of GPI-ICAM-1 or anti-κ antibody at the lipid bilayers by immunofluorometric assay using anti-ICAM-1 or anti-rat-IgG antibodies, respectively; we obtained the standard values from microbeads with distinct calibrated IgG-binding capacities (Bangs Laboratories). Before imaging, membranes were coated with murine recombinant CXCL13 (100 nM, Peprotech; 20 min, RT).

Unlabeled or violet-tracer-labeled (0.1 μM, 10 min, 37°C; Molecular Probes) primary B cells (2 × 10^6^) were injected into the warmed chamber (37°C) and imaging was begun. When indicated, B cells were pretreated with the Btk inhibitor ibrutinib (50 nM, 1 h, 37°C; IC_50_ 0.5 nM; Selleckchem) or acalabrutinib (ACP-196) (500 nM or 10 nM dose, 1 h, 37°C; IC_50_ 5 nM; Selleckchem); inhibitors were maintained during the assay. Confocal fluorescence (1 μm-optical section), differential interference contrast (DIC), and IRM images were acquired every 30 s for 10–20 min; consecutive videos were acquired when needed. For Ca^2+^ flux measurements, B cells were labeled with Fluo-4FF (1 μM, 30 min, RT; Molecular Probes), injected immediately into the warmed FCS2 chamber, and imaged every 10 s for 15 min at low quality to speed up acquisition. Assays were performed in chamber-buffer (PBS, 0.5% FCS, 0.5 g/l D-glucose, 2 mM MgCl_2_, 0.5 mM CaCl_2_). Images were acquired on an Axiovert LSM 510-META inverted microscope with a 40X oil immersion objective (Zeiss).

### Immunofluorescence

Primary B cells were in contact with ICAM-1/CXCL13 membranes containing tethered surrogate antigen for the specified times in each case (10 min for vinculin, talin, WASP, Btk, p-Btk and p-PLCγ2), fixed with 4% paraformaldehyde (10 min, 37°C), permeabilized with PBS/0.1% Triton-X100 (5 min, RT), blocked with PBS/2% FCS/2%BSA (overnight, 4°C), and stained with AlexaFluor 647 phalloidin (Molecular Probes) plus the following antibodies. Rabbit anti-WASP (a kind gift of I.M. Antón, CNB-CSIC, Spain) or anti-Btk (D3H5; Cell Signaling) plus AlexaFluor 488 goat anti-rabbit IgG (Southern Biotechnology); mouse anti-talin or -vinculin (clones 8d4 and hVIN-1, respectively; Sigma), AlexaFluor 488 mouse anti-phospho-PLCγ2 (Y759; clone K86-689.37; BD Biosciences) or AlexaFluor 488 anti-phospho-Btk (Y223; clone N35-86; BD Biosciences) plus FITC goat anti-mouse IgG1 (BD Biosciences). To combine PIP_2_ detection with a plasma membrane fluorescent probe, B cells were labeled with CellVue Claret Far Red probe (Sigma) following kit instructions, then settled on su-Ag-containing artificial membranes for 2 min, fixed them with 4% PFA (10 min, 37°C), permeabilized with 0.2% Tween-20 (5 min, RT), blocked as above and stained with mouse anti-PIP_2_ (clone ab2335, Abcam) plus FITC goat-anti-mouse IgG2b (BD Biosciences) (30 min per step, RT). FCS2 chambers were imaged by confocal fluorescent microscopy as above.

### Cell conjugates and activation assays

To prepare pseudo-APC, silica beads (5 × 10^6^; 5 μm diameter; Bangs Laboratories) were washed in distilled water (5,000 rpm, 1 min, RT), incubated with 20 μl DOPC liposomes containing GPI-linked ICAM-1 (200 molec/μm^2^) and biotin-lipids (20 or 100 molec/μm^2^) (10 min, RT), washed twice with chamber-buffer, and then blocked, the surrogate antigen tethered and coated with CXCL13 as for planar artificial membranes. For cell activation assays, freshly isolated B cells (2 × 10^5^) were co-cultured with pseudo-APC, unloaded or loaded with surrogate antigen at a 1:1 ratio (20 h) in flat-bottom p96 plates, collected, and analyzed by flow cytometry. To measure cell proliferation, B cells were labeled with violet-tracer (1 μM, 10 min, 37°C), washed with complete RPMI/10% FCS, and co-cultured with pseudo-APC, unloaded or loaded with surrogate antigen at specified ratios, with recombinant murine IL-4 (10 ng/ml, Peprotech) (72 or 96 h). Cells were collected, stained for APC-conjugated CD19, and analyzed for violet-tracer dilution in a LSR-II cytometer. When required, B cells were pretreated with the Btk inhibitors (ibrutinib, acalabrutinib) at the specified doses 1 h before adding the pseudo-APC to the culture.

### Imaging data analysis

The frequency of IRM or su-Ag central cluster per imaged field was estimated as [n° of B cells showing IRM contact or a central antigen aggregate or cSMAC formed (with fluorescence antigen)/total n° of B cells (estimated by DIC)] × 100, using FiJi (NIH) software. One-micrometer-optical section confocal images were acquired at the contact plane/IS plane or mid-cell plane; we used the IRM confocal image to focus on the B cell/artificial membrane contact plane and to define the IS plane. Imaris 7.0 software (Bitplane) was used for qualitative and quantitative analysis of fluorescence signals at distinct cell planes, as well as for cell contact area (IRM area) and antigen aggregate area measurements. To set up the background of FL intensity signal, we used the FL signal of the artificial membrane in each case.

To apply statistics analysis to the Ca^2+^ influx curves, we followed an approach used in pharmacokinetic studies. We calculated the area under de curve (AUC) for each cell in each condition (WT, Xid, untreated or treated with Btk inhibitors) and then compared the obtained AUC values with those of the control condition (untreated WT). To obtain the AUC value per cell, we sectioned the area in three trapezoids, calculated the area for each one, and added them up.

### Western blot analysis

Freshly isolated primary B cells (5 × 10^6^) were cultured on a p48 plate in depletion medium (0.5 ml complete RPMI, 1 h), in the presence of 50 nM ibrutinib or 500 nM acalabrutinib when needed, and then stimulated in Ab-coated (goat anti-mouse IgM, μ-specific; Jackson ImmunoResearch) p48 plate for the indicated times. Wells were pre-coated with 5 μg/ml Ab in PBS (1 h, 37°C), washed and used. Cells were lysed in lysis buffer (10 mM Tris-HCL pH 7.4, 150 mM NaCl, 1 mM EDTA)/1% Triton-X100 with protease and phosphatase inhibitors (Roche; 30 min, 4°C). Lysates were centrifuged (14,000 rpm, 30 min, 4°C), supernatants collected and stored at −80°C. Total protein was quantified with the Micro BCA Protein Assay kit (Thermo Scientific), separated by SDS-PAGE and transferred to PVDF membranes (BioRad). Blots were blocked with TBS-T (10 mM Tris-HCl pH 8.0, 150 mM NaCl, 0.1% Tween-20)/5% BSA (1 h, RT), and incubated with rabbit anti-cofilin, -phospho-cofilin (S3),-phospho-ERK1/2 (Y202/Y204), -phospho-PKCα/β (T638/641), -phospho-Akt (S473) (Cell Signaling), or loading control mouse anti-α-tubulin (clone DM1A; Sigma) (overnight, 4°C), followed by horseradish peroxidase-conjugated secondary antibodies (DAKO; 1 h, RT). The signal was detected with the enhanced chemiluminiscence detection system (ECL; GE Healthcare). Signal intensity values in arbitrary units (AU) for each protein were quantified using FiJi (NIH) software, normalized to tubulin.

For Btk and Tec protein detection, freshly isolated primary B cells (5 × 10^6^) were lysated as above. Total protein was quantified, separated by SDS-PAGE and transferred to PVDF membranes. Membranes were incubated with rabbit anti-Btk (D3H5) or anti-Tec (both from Cell Signaling), followed by horseradish peroxidase-conjugated secondary antibodies; ECL was used for signal detection, as above.

### Rac GTPase activity assays

Rac GTPase activity was determined in B cell lysates using the G-LISA Rac kit (BK125; Cytoskeleton). Freshly isolated primary B cells (5 × 10^6^) were arrested and stimulated in Ab-coated p48 plates for indicated times, as above. One volume of ice-cold PBS was added to terminate stimulation, eliminated by vacuum, and G-LISA kit lysis buffer was added (100 μl/well). Lysis and GTPase activity were measured following G-LISA kit instructions.

### Flow cytometry

B cells were stained with fluorochrome-conjugated (FITC, PE, or APC) rat anti-mouse IgD, CD19, CD69, or CD86, biotinylated rat anti-mouse CD25 plus PE-conjugated avidin (BioLegend), and DyLight-649-conjugated Fab fragment goat anti-mouse IgM, μ-specific (Jackson ImmunoResearch) (30 min, 4°C). Samples were acquired in a LSR-II cytometer (Beckman Coulter) and data analyzed with FlowJo software (BD Biosciences).

For cell sorting purposes, total splenocytes from wild type mice were labeled with Cy3-conjugated Fab goat anti-mouse IgM, μ-chain specific (30 min, 4°C) and separated in IgM^hi^ and IgM^med^ B cells using a FACSAria Fusion flow cytometer. Fab fragments anti-IgM do not crosslink the BCR and thus do not trigger B cell activation.

### Statistical analysis

Graphs and statistical analyses were performed using GraphPad Prism 6.0f software. Two-tailed unpaired Student's *t*-test was applied, always related to WT B cell values; ^*^p < 0.05; ^**^p < 0.01; ^***^p < 0.001; ^****^p < 0.0001.

## Results

### Distinct roles for Btk shuttling/scaffold and kinase activities in B cell IS formation

To interfere with the shuttling/scaffold and kinase functions of Btk, we used primary B cells isolated from CBA/N (Xid) mice, which bear a point mutation at the Btk PH domain that affects PIP_3_ binding and thus, Btk recruitment to the plasma membrane (23). The IgM/IgD expression profile and Btk protein levels of isolated Xid compared to wild type (WT) B cells from distinct genetic backgrounds are shown in (Supplementary Figures 1A,B). To alter only Btk-kinase activity, we treated primary B cells with the inhibitor ibrutinib (PCI-32765) (24); we tested several doses and selected one (50 nM) that inhibited kinase function without affecting cell survival (Supplementary Figures 1C,D).

**Figure 1:**
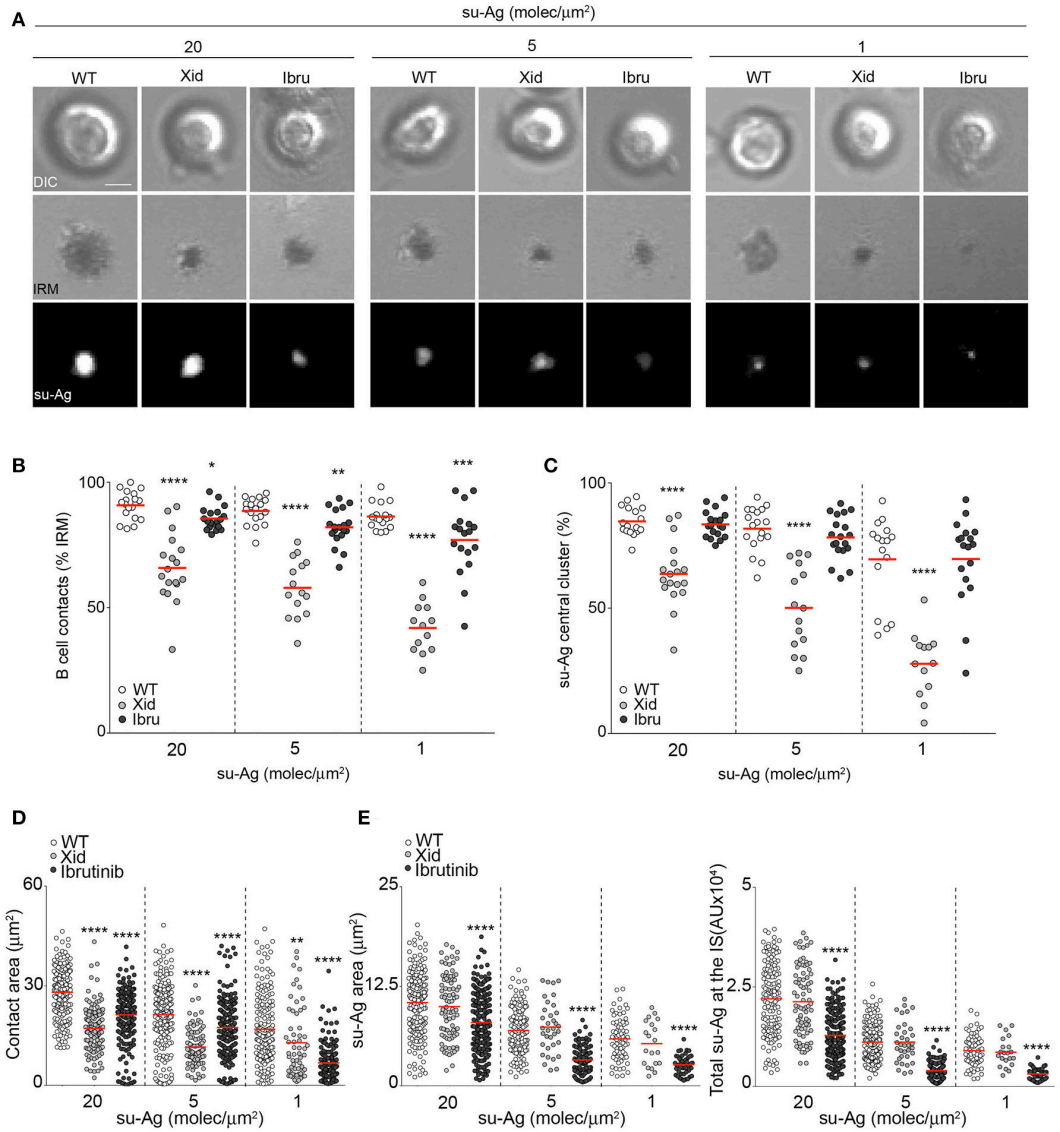
Btk regulates distinct aspects of B cell IS formation. **(A)** DIC, IRM and fluorescence su-Ag images at the contact plane of representative IS-forming WT, Xid and Ibru-B cells. Bar, 2 μm. **(B)** Frequencies of B cell contacts (IRM^+^ cells) and **(C)** su-Ag central cluster formation. Values of **(D)** contact area, **(E)** su-Ag aggregate area (cSMAC; left) and total su-Ag fluorescence (FL) (in arbitrary FL units, AU; right) for B cells with established IS in each case. Each dot in **(B)** represents a single image field, and in **(C,D)** a single cell. Data of a representative experiment are shown in **(B–D**) (*n* = 4). ^*^p < 0.05; ^**^p < 0.01; ^***^p < 0.001; ^****^p < 0.0001 by Student's *t*-test with WT in each case.

To monitor B cell IS formation, we used a biomimetic model combined with real-time confocal microscopy (11). This model recreates the APC surface by assembling planar artificial lipid bilayers containing glycosylphosphatidylinositol (GPI)-linked ICAM-1, a CXCL13 chemokine coating, and tethered surrogate antigen (anti-κ light chain antibody; su-Ag) at different densities. We allowed freshly isolated WT, Xid, and ibrutinib-treated WT (Ibru) B cells to settle on the artificial membranes (10 min), and then imaged them to evaluate their ability to form the IS [estimated by two criteria: (1) detection of a central cluster of fluorescent su-Ag and (2) detection of cell contact with the artificial membrane using interference reflection microscopy, IRM] (Figure 1A). Cell contact and su-Ag central cluster frequency values for WT B cells varied depending on su-Ag density, as expected (100–50%); Ibru-B cells were hardly affected, while Xid B cells had significantly reduced the capacity to establish the IS compared to WT (60–30%) (Figures 1B,C). We analyzed pSMAC/cSMAC assembly in those B cells with established IS. Using IRM, we measured the B cell contact area with the artificial membrane; this area represents the sum of the pSMAC plus cSMAC areas. Both Xid and Ibru-B cells showed smaller contact areas than controls (Figures 1A,D). Quantification of the area and total quantity of su-Ag aggregated at the cSMAC (both estimated by fluorescence) indicated that Ibru-B cells had smaller cSMAC and accumulated less su-Ag at the IS than controls; values for area and total su-Ag aggregation in Xid B cells were similar to those for WT (Figures 1A,E). Ibrutinib treatment of Xid B cells (Xid-Ibru) resulted in reduced su-Ag area and aggregation compared with untreated Xid B cells; contact area values were also smaller than for Xid (Supplementary Figure 1E). Btk membrane recruitment appeared then to regulate the B cell ability to trigger IS formation, evaluated as the capacity to make contact with the artificial membrane and to form a su-Ag central cluster; the Btk shuttling/scaffold activities seemed more relevant than the kinase function on that. In addition, IS-forming B cells with impaired Btk shuttling/scaffold functions showed defects in the pSMAC domain while Btk-kinase inhibition decreased the antigen quantity that accumulated at the cSMAC. WT B cells isolated from CBA/Ca and C57BL/6 mice presented equal results (data not shown).

### Btk kinase activity controls the B cell spreading phase during IS formation

**Figure 2:**
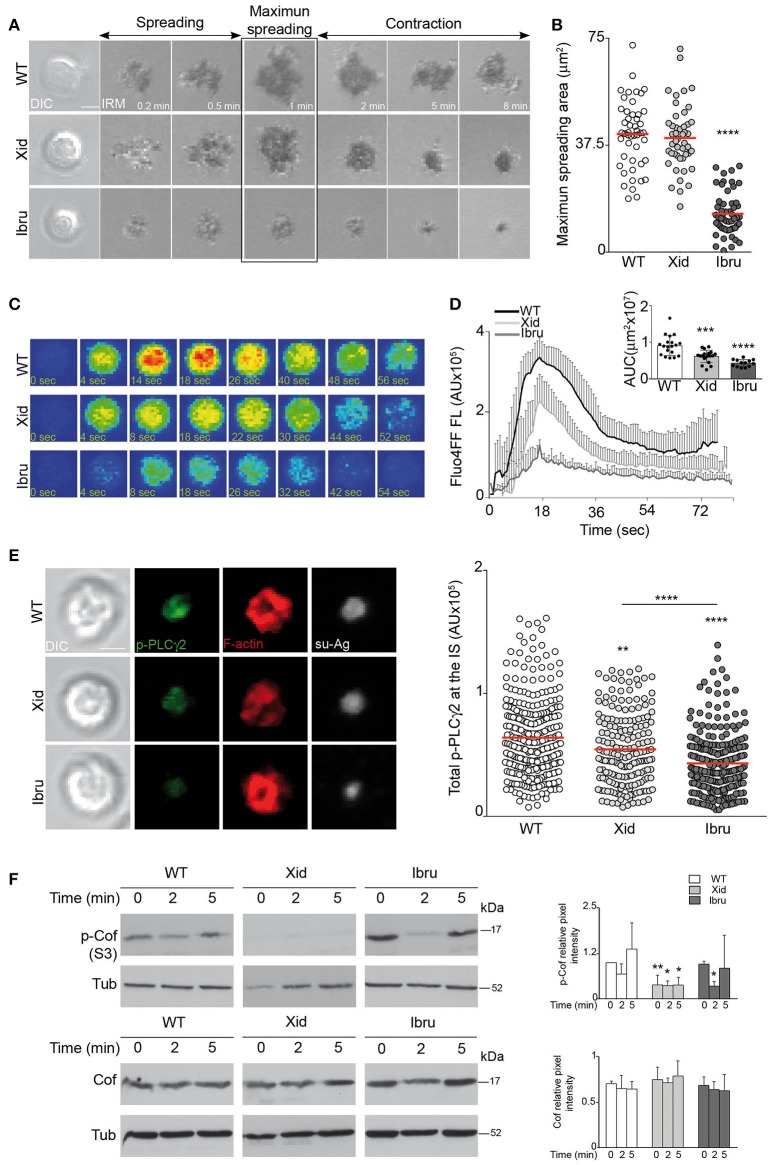
Antigen gathering is controlled by Btk-kinase activity. **(A)** DIC and IRM images for representative WT, Xid, and Ibru-B cells over time while forming the IS; antigen, 20 molec/μm^2^. Spreading and contraction phases are indicated; maximum cell spreading time is highlighted. **(B)** Maximum spreading area values; each dot represents a single cell. **(C)** Fluo4FF-labeled WT, Xid and Ibru-B cells as in **(A)** were monitored for Ca^2+^ influx. Fluorescence Fluo4FF images of representative B cells over time are shown. **(D)** Values of total Fluo4FF FL (in AU) over time; data shown as the mean ± SD of 20 B cells/condition. Inner bars-graph, AUC values per B cell and per condition; each dot is a cell. **(E)** DIC and fluorescence images of phosphorylated PLCγ2 (p-PLCγ2; green), F-actin (red) and antigen (Ag; white) for representative IS-forming B cells, fixed at 10 min; antigen, 20 molec/μm^2^. Right, values of total p-PLCγ2 FL (in AU) at the IS. **(F)** Immunoblot of WT, Xid, and Ibru-B cells stimulated with Ab-coated plates, probed with specified antibodies. Right, quantification of phosphorylated-cofilin (p-Cof, S3) and cofilin (Cof) intensity normalized to tubulin (Tub; loading control) by densitometry at each time point. Data for a representative experiment are shown in **(B)** (*n* = 4), **(D)**, and **(E)** (*n* = 3); data in F are the mean ± SD of three experiments (*n* = 3). Bar 2 μm. ^*^p < 0.05; ^**^p < 0.01; ^***^p < 0.001; ^****^p < 0.0001 by Student's *t*-test with WT in each case.

Tethered antigen recognition through the BCR triggers a two-step process that leads to IS formation, an initial cell-spreading phase (first 2 min of B cell-APC interaction) followed by the cell-contraction phase (in the subsequent 5 min). The magnitude of B cell spreading is dictated by BCR signaling strength and largely determines the antigen quantity that accumulates at the cSMAC (25). We used IRM to evaluate the maximum spreading area of WT, Xid and Ibru-B cells in contact with su-Ag-containing artificial membranes. Ibru-B cells showed a strong reduction in maximum spreading area compared to WT, whereas Xid B cells had normal BCR-triggered spreading capacity (Figures 2A,B).

PLCγ2 activation by Btk triggers Ca^2+^ mobilization; both PLCγ2 and Ca^2+^ signals are involved in the spreading phase (6, 26). Using real-time fluorescent microscopy, we measured Ca^2+^ mobilization during IS formation in WT, Xid and Ibru-B cells preloaded with the Ca^2+^-sensitive fluorescent probe Fluo4FF. BCR recognition of tethered su-Ag triggered a peak followed by sustained Ca^2+^ influx in WT B cells. In Xid B cells, both the peak and sustained Ca^2+^ influx were lower than controls, whereas Ca^2+^ influx was barely detectable in Ibru-B cells (Figures 2C,D). To statistically compared the curves, we applied an approach used in pharmacokinetic studies. We calculated the area under the curve (AUC) for each cell in each condition (WT, Xid, Ibru), and then compared the AUC values obtained for Xid or Ibru-B cells with those for controls (Figure 2D). We analyzed Btk-dependent PLCγ2 phosphorylation at residue Y759 (p-PLCγ2) at the synapse by immunofluorescence (IF); for that, B cells were in contact with artificial membranes for 10 min, fixed and stained. Ibru-B cells had a stronger reduction in p-PLCγ2 levels at the IS than Xid B cells compared to controls (Figure 2E). PLCγ2/Ca^2+^ influx promotes F-actin rearrangement and cell spreading by activating the actin-severing protein cofilin through transient dephosphorylation at S3 (26). We analyzed cofilin phosphorylation levels (p-Cof) in lysates of B cells stimulated on anti-μ HC antibody (Ab)-coated plates. WT B cells showed decreased p-Cof levels at the time of cell spreading (2 min) and then recovered (contraction phase, 5 min; Figure 2F). Basal p-Cof levels were low in Xid B cells and barely changed after BCR stimulation, whereas Ibru-B cells underwent stronger transient cofilin activation than controls (Figure 2F). Data indicated that Btk-kinase activity determines the quantity of antigen accumulated at the cSMAC by regulating initial B cell spreading through the PLCγ2/Ca^2+^ influx axis; cofilin appears not to be involved. Xid-Btk reduced PLCγ2 activation and Ca^2+^ influx, but with no effects on the spreading phase.

### Vinculin recruitment and F-actin levels at the IS are impaired in Xid B cells

**Figure 3:**
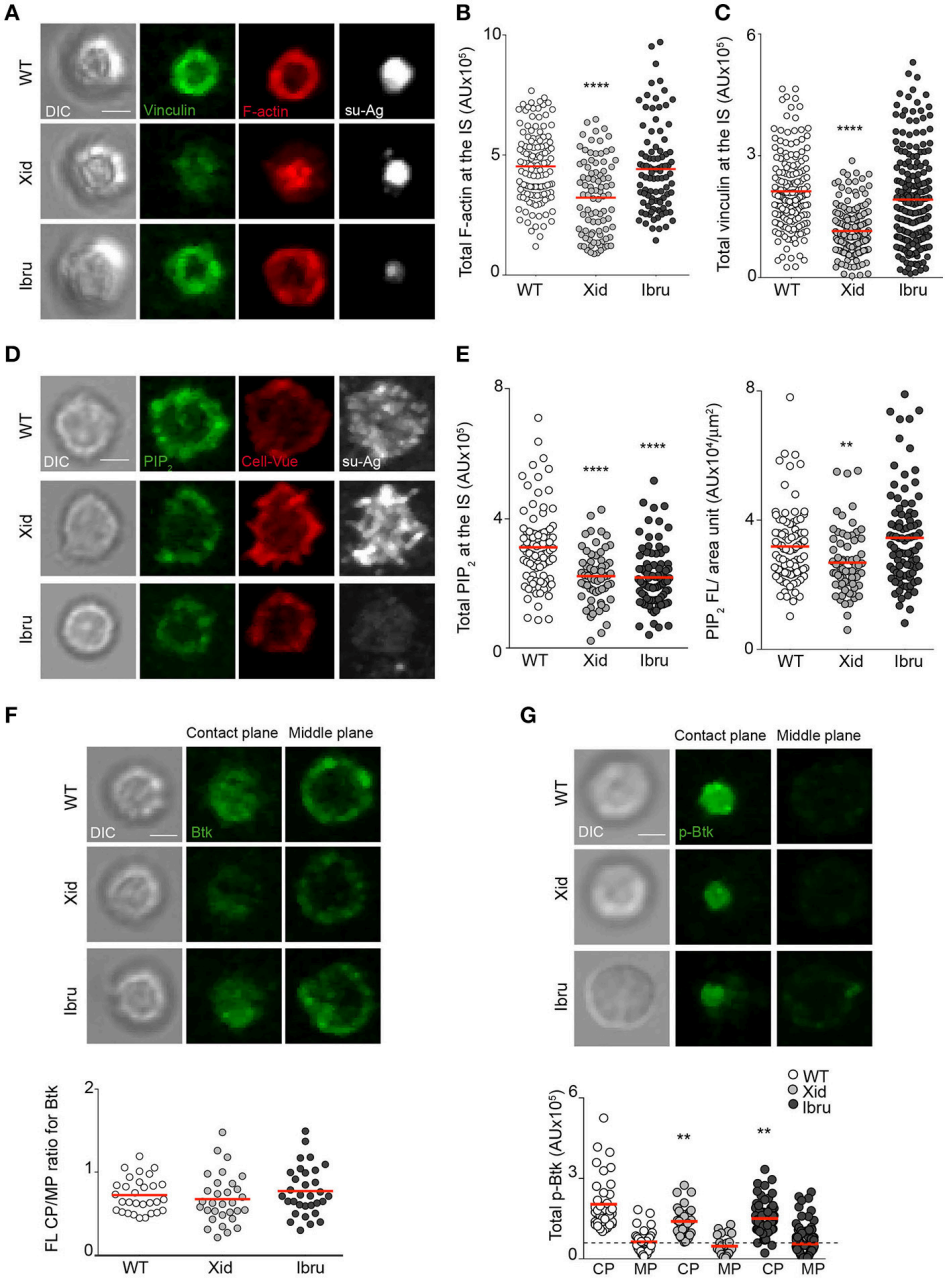
Btk shuttling/scaffold activity regulates vinculin recruitment and F-actin polymerization at the B cell IS. **(A)** DIC and fluorescence images of vinculin (green), F-actin (red) and su-Ag (white) for representative IS-forming WT, Xid, and Ibru-B cells, fixed at 10 min; antigen, 20 molec/μm^2^. Values of **(B)** total F-actin FL and **(C)** total vinculin FL (in AU) at the IS. **(D)** DIC and fluorescence images of PIP_2_ (green), CellVue (red) and su-Ag (white) for representative WT, Xid and Ibru-B cells, fixed at 2 min; antigen, 20 molec/μm^2^. **(E)** Quantification of total PIP_2_ FL at the B cell-artificial membrane contact plane (left) and of PIP_2_ FL/area unit (right). **(F)** DIC and fluorescence images for Btk (green) at the contact and mid-cell planes for representative WT, Xid and Ibru-B cells. Bottom, ratio of total Btk fluorescence at the contact plane (CP) to that at the mid-cell plane (MP) for each B cell type. **(G)** DIC and fluorescence images for phosphor-Y223-Btk (p-Btk; green) at the contact and mid-cell planes for representative WT, Xid, and Ibru-B cells. Bottom, quantification of total p-Btk fluorescence at the CP and MP; dotted line, basal p-Btk signal in absence of su-Ag. Each dot represents a single cell in **(B,C,E–G)**. Data from a representative experiment are shown in **(B–G)** (*n* = 2). Bar 2 μm. ^**^p < 0.01; ^****^p < 0.0001 by Student's *t*-test with WT in each case.

To study pSMAC molecular organization inside B cells, we used IF and analyzed vinculin recruitment and distribution as well as F-actin levels in WT, Xid, and Ibru-B cells forming IS. Vinculin localized with F-actin in the pSMAC, forming a ring-shaped structure surrounding the antigen cluster in the majority of WT B cells (Figure 3A), as previously reported (12); vinculin/F-actin distribution in Ibru-B cells was similar to controls. In Xid B cells, vinculin and F-actin collapsed at the center, together with the su-Ag cluster (Figure 3A). To estimate total vinculin recruited and F-actin at the IS, we measured total fluorescence intensity at the synapse. The values for Ibru-B cells were comparable to those for WT, whereas Xid B cells recruited significantly less vinculin and have less F-actin at the IS than controls (Figures 3B,C). Ibrutinib treatment did not modify vinculin and F-actin defects at the Xid B cell IS (Supplementary Figures 2A,B). Xid B cells are enriched in IgM^hi^ immature populations (Supplementary Figure 1A) due to deficiencies in B cell maturation (1). We studied F-actin accumulation and ring-assembly at the IS of IgM^hi^ compared to IgM^med^ WT B cells by IF. We isolated both B cell populations from splenocytes using a cell-sorter flow cytometer (Supplementary Figure 2C), and then settled them on su-Ag-containing artificial membranes, fixed and stained them. IgM^hi^ B cells had more F-actin than IgM^med^ B cells at the IS (Supplementary Figure 2D). We evaluated the B cell ability to distribute the F-actin in well-defined rings at the IS, and found lower frequency values for IgM^hi^ compared to those for IgM^med^ B cells (Supplementary Figure 2D). These results suggested that Xid B cell defects on F-actin at the IS were not due to their immature phenotype.

The defects in pSMAC molecular organization found in Xid B cells led us to analyze talin and WASP proteins, both involved in integrin-ring assembly and actin polymerization at the pSMAC; they showed altered distribution and total quantity recruited at the IS of Xid B cells compared to controls (Supplementary Figures 2E,F). Local PIP_2_ production at the plasma membrane is needed for adhesion site assembly and actin dynamics (27). A wave of local PIP_2_ synthesis takes place during B cell IS formation (12). As Btk regulates PIP_2_ production through the PIP5KI shuttling (4), we evaluated PIP_2_ levels at the synapse of Xid B cells compared to controls by IF. We measured PIP_2_ levels at early times (at 2–3 min) during IS formation, when PIP_2_ production is highest, to facilitate detection by IF. B cell labeling with a fluorescent membrane probe (CellVue) allowed us to evaluate plasma membrane topology/density at the imaged plane. Xid B cells had lower PIP_2_ levels than WT at the IS, measured as total fluorescence (FL) or normalized it to the spread-membrane area estimated by CellVue FL (PIP_2_ FL/area unit, μm^2^) (Figures 3D,E). Similarly, we analyzed PIP_2_ in Ibru-B cells and found reduced total FL values compared to WT; when normalized to the spread-membrane area, diminished in Ibru-B cells (Figure 2B), values where comparable to those for control cells (Figures 3D,E). Rac GTPases control F-actin polymerization at the IS (13); we evaluated Rac activation in lysates of B cells stimulated with Ab-coated plates at early times of IS formation (first 2 min), when a burst of F-actin polymerization is needed for the initial cell spreading phase. We found no significant differences in Rac activation for Xid or Ibru-B cells compared to controls (Supplementary Figure 2G). Data thus suggested that Btk shuttling/scaffold activities control pSMAC molecular assembly; regulation of PIP_2_ production at the plasma membrane seems to be involved.

We evaluated Btk recruitment to the IS by IF, fixing B cells 10 min after contact with the su-Ag-containing artificial membrane. We imaged the contact and mid-cell planes by confocal microscopy, measured the total FL values in each plane and calculated the ratio (contact/mid-cell plane); ratio values >1 indicated protein recruitment to the contact plane. We obtained ratio values <1 for WT B cells, suggesting no substantial Btk enrichment at the IS; Xid and Ibru-B cells showed ratio values similar to controls (Figure 3F). We then investigated active Btk protein by using an anti-phosphorylated-Y223 antibody. WT B cells accumulated phosphorylated Btk (p-Btk) at the contact plane (Figure 3G); p-Btk levels were lower in Xid and Ibru-B cells (Figure 3G).

### Defects in Btk membrane-recruitment or kinase activity affects MTOC polarization, B cell activation, and proliferation

**Figure 4:**
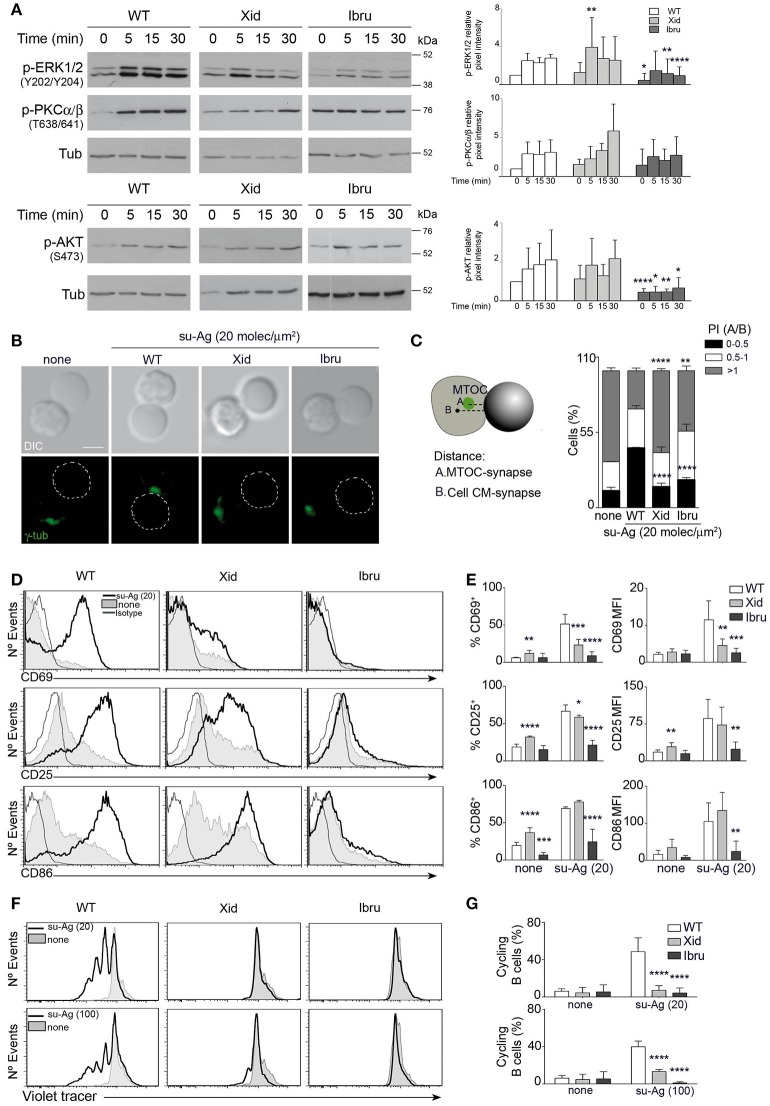
Btk functional deficiency alter MTOC polarization, B cell activation and proliferation. **(A)** Immunoblot of WT, Xid, and Ibru-B cells stimulated with Ab-coated plates, probed with the specified antibodies. Quantification of phosphorylated ERK1/2 (p-ERK1/2, Y202/Y204), phosphorylated PCKα/β (p-PKCα/β, T638/641) and phosphorylated Akt (p-Akt, S473) intensity normalized to tubulin (Tub; loading control) by densitometry at each time point; right. **(B)** DIC and fluorescence γ-tubulin (γ-tub; green) images are shown of representative B cell/pseudo-APC conjugates, fixed at 30 min, for each indicated condition. Dashed circle, pseudo-APC; Bar, 2 μm. **(C)** Frequency of B cells as in B, in the specified polarity index (PI) groups. The PI per B cell was estimated as the ratio of A and B distances (explicative diagram, left); data shown as mean ± SD of 90 B cells in each case. **(D–E)** B cells were co-cultured with pseudo-APC, unloaded (none) or su-Ag-loaded (20 molec/μm^2^; su-Ag-20) at 1:1 ratio, 20 h. **(D)** Representative profiles of CD69, CD25 and CD86 for each case. **(E)** Frequencies of B cells expressing CD69, CD25 or CD86 (left) and mean fluorescence intensity (MFI) values for these markers (right) in each condition and for each B cell type. **(F–G)** Violet-labeled B cells were co-cultured with pseudo-APC, unloaded (none) or su-Ag-loaded (20 molec/μm^2^, su-Ag-20, ratio 1:5; 100 molec/μm^2^, su-Ag-100, ratio 1:1), with IL-4, 96 h. **(F)** Representative profiles of violet-tracer in each case. **(G)** Frequencies of cycling B cells (with diluted violet-tracer level) in each condition and for each B cell type. Data in **(A)** are the mean ± SD of three experiments (*n* = 3); data in **(C)** are the mean ± SD of two experiments (*n* = 2), and in **(E,G)** are the mean ± SD of three experiments (*n* = 3). ^*^p < 0.05; ^**^p < 0.01; ^***^p < 0.001; ^****^p < 0.0001 by Student's *t*-test with WT in each case.

We evaluated the alterations caused by Xid-Btk or Ibru-treatment in BCR-triggered B cell activation events. We initially analyzed activation of early signaling molecules in lysates of WT, Xid and Ibru-B cells stimulated on Ab-coated plates. Ibru-B cells showed low MAPK ERK1/2 and Akt activation levels and almost no detectable activation kinetic of classic PKCα/β compared to controls. Xid B cells activated ERK1/2, Akt, and PKCα/β similarly to WT (Figure 4A).

B cell function depends on its antigen extraction, processing and presentation abilities. MTOC repositioning to the IS supports the vesicle/membrane trafficking required for that (20, 28). We examined MTOC polarization to the synapse of Xid and Ibru-B cells compared to WT. To facilitate this evaluation, we assembled the artificial lipid bilayers on the surface of silica beads (5 μm diameter). The artificial membranes were functional (fluid; not shown) and, like the artificial planar membranes, contained GPI-linked ICAM-1, CXCL13 coating and tethered su-Ag. B cells formed conjugates with these pseudo-APC and established the IS (Supplementary Figure 3A); at 30 min post-contact, B cell-bead conjugates were fixed, permeabilized and stained for γ-tubulin (Figure 4B). For each B cell, we measured the MTOC distance to the IS (A) and normalized it to the distance from the cell center of mass to the synapse (B) to obtain the polarization index (PI; ratio A/B) (Figure 4C). In the majority of WT B cells in contact with su-Ag-bearing pseudo-APC, the MTOC polarized to the IS (72%; PI < 1), at difference from none su-Ag. MTOC polarization to the IS was altered in Btk-impaired B cells, more severely in Xid B cells (40% with PI < 1) than in Ibru-B cells (56% with PI < 1).

We studied BCR-triggered late activation events such as the expression of the surface markers CD69, CD25 and CD86 and cell proliferation. B cells were co-cultured for 20 h at a 1:1 ratio with pseudo-APC, unloaded or su-Ag-loaded, then collected and analyzed by flow cytometry (Figures 4D,E). In the presence of su-Ag, almost all WT B cells showed increased CD69, CD25 and CD86 levels. Xid B cells had lower CD69^+^CD25^+^ B cell frequencies and levels of both markers than controls; CD86 expression was unaffected. Ibru-B cells exposed to su-Ag showed no increase in the levels of these markers. For proliferation assessment, violet-tracer labeled B cells were co-cultured with pseudo-APC, unloaded or loaded with su-Ag at various densities (20 or 100 molec/μm^2^) and at different ratios per B cell (5:1 or 1:1, respectively), in the presence of IL-4. After 96 h, we estimated B cell proliferation by measuring violet-tracer labeling dilution in a flow cytometer. In contrast to WT, Xid and Ibru-B cells did not proliferate in response to su-Ag (Figures 4F,G). In both activation and proliferation assays, Xid and Ibru-B cell viability was not markedly compromised compared to controls in absence of su-Ag (Supplementary Figures 3B,C).

### The second-generation Btk inhibitor acalabrutinib (ACP-196) alters B cell IS formation similar to ibrutinib

**Figure 5:**
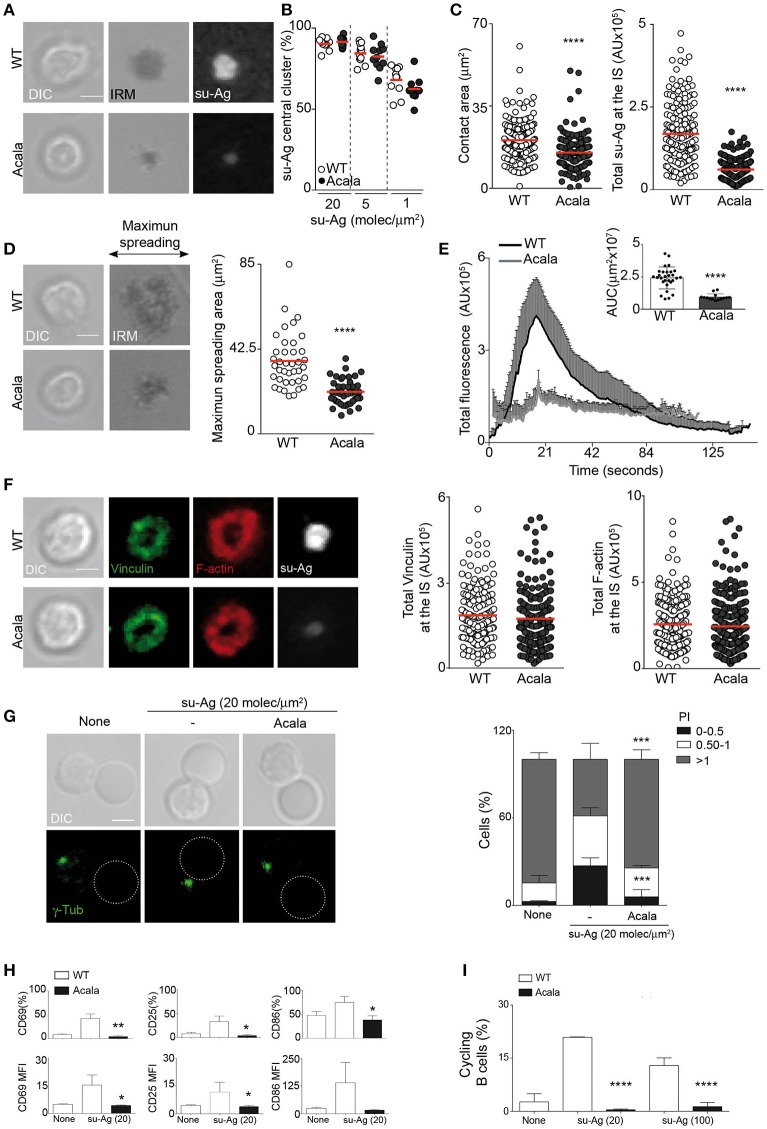
Acalabrutinib affects su-Ag-triggered IS formation, cell activation and proliferation. **(A)** DIC, IRM, and fluorescence su-Ag images at the contact plane of representative IS-forming WT B cells, untreated or treated with Acala (500 nM); su-Ag, 20 molec/μm^2^. **(B)** su-Ag central cluster formation frequency in presence of distinct su-Ag densities. **(C)** Values of contact area (estimated by IRM; left) and total su-Ag FL (in AU; right) for IS-forming B cells; su-Ag, 20 molec/μm^2^. **(D)** DIC and IRM images of representative untreated or Acala-treated B cells at the time of maximum cell spreading during IS formation. Right, maximum spreading area values (estimated by IRM). **(E)** Fluo4FF-labeled untreated or Acala-treated B cells were monitored for Ca^2+^ influx; total Fluo4FF FL values (in AU) over time are shown; data shown as mean ± SD of 20 B cells/condition. Inner bars-graph, AUC values per B cell and per condition; each dot is a cell. **(F)** DIC and fluorescence images of vinculin (green), F-actin (red), and su-Ag (white) for representative IS-forming B cells, untreated or Acala-treated, fixed at 10 min; su-Ag, 20 molec/μm^2^. Right, values of total vinculin and F-actin FL (in AU) at the IS. Bar, 2 μm. Each dot in **(B)** represents a single image field, and in **(C–D,F)**, a single cell. Data from a representative experiment are shown in **(B–F)** (*n* = 3). **(G)** DIC and fluorescence γ-tubulin (γ-tub; green) images are shown of representative untreated or Acala-treated B cell/pseudo-APC conjugates. Dashed circle, pseudo-APC; Bar, 2 μm. Right, frequency of B cells in the specified polarity index (PI) groups. The PI per B cell was estimated as explained in Figure 4C; data shown as mean ± SD of 80 B cells in each case. **(H)** Untreated or Acala-treated B cells were co-cultured with pseudo-APC, unloaded (none) or su-Ag-loaded (20 molec/μm^2^; su-Ag-20) at 1:1 ratio, 20 h. Frequencies of B cells expressing CD69, CD25 or CD86 (top) and MFI values for these markers (bottom) in each condition are shown. **(I)** Violet-labeled untreated and Acala-treated B cells were co-cultured with pseudo-APC, unloaded (none) or su-Ag-loaded (20 molec/μm^2^, su-Ag-20, ratio 1:5; 100 molec/μm^2^, su-Ag-100, ratio 1:1), with IL-4, 72 h. Frequencies of cycling B cells in each condition are shown. Data in **(B)** are the mean ± SD of four experiments (*n* = 4); data of a representative experiment are shown in **(C–F)** (*n* = 3); data in **(G)** is the merge of two experiments and in **(H,I)** of three experiments (*n* = 3). Each dot in **(B)** represents a single image field, and in **(C–D,F)**, a single cell. ^*^p < 0.05; ^**^p < 0.01; ^***^p < 0.001; ^****^p < 0.0001 by Student's *t*-test with WT in each case.

The untoward effects of Ibrutinib treatment observed in the clinic, probably due to off-target effects on other kinases, led to the development of more selective Btk inhibitors. Acalabrutinib (ACP-196) is an irreversible second-generation inhibitor with higher potency and specificity for Btk, and thus reduced off-target activity (29). To strength our studies on the Btk kinase role for B cell IS formation and to discard possible bystander effects of Ibru-treatment, we performed experiments treating B cells with acalabrutinib (Acala). B cell pre-treatment with 500 nM Acala decreased BCR-triggered ERK1/2 activation without altering cell viability in culture (Supplementary Figures 4A,B). The studies performed on IS formation using the artificial membranes showed that Acala-B cells behaved similar to Ibru-B cells. The frequencies of su-Ag central cluster formation were comparable to those for untreated B cells at distinct su-Ag densities (Figures 5A,B). Acala-B cells had however lower values of contact area (IRM area) and total su-Ag accumulated at the IS than controls (Figure 5C). Decreased su-Ag aggregation correlated with diminished maximum spreading area values at early time-points during IS formation as well as hardly detectable Ca^2+^ influx (Figures 5D,E). Studies on vinculin/F-actin accumulation and distribution at the synapse by IF showed no differences between Acala- and WT B cells (Figure 5F).

We investigated Acala treatment effects on MTOC polarization to the IS. We allowed B cells to form conjugates with pseudo-APC, unloaded or su-Ag-loaded, and then analyzed γ-tub by IF, as previously explained. Acala treatment significantly reduced MTOC polarization to the IS (Figure 5G). Next, we co-cultured untreated or Acala-treated B cells with pseudo-APC in absence or presence of su-Ag, and evaluated cell activation and proliferation (using violet-tracer-labeled B cells) by flow cytometry at 20 and 72 h, respectively. CD69, CD86 and CD25 surface expression triggered by su-Ag was almost abolished in Acala-B cells (Figure 5H); cell proliferation was also inhibited (Figure 5I). These results mirrored those obtained for Ibru-B cells.

### B cells lacking Btk protein have no defects on IS formation

**Figure 6:**
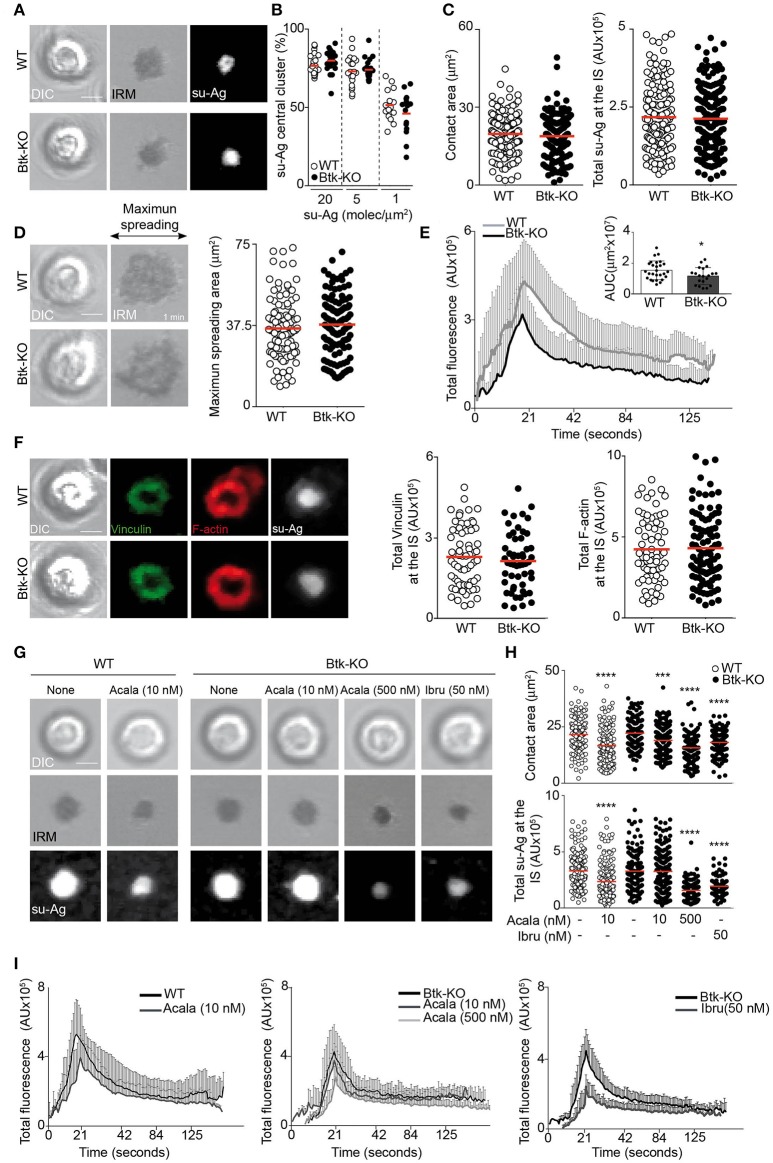
B cells lacking Btk have no defects in IS formation. **(A)** DIC, IRM and fluorescence su-Ag images at the contact plane of representative IS-forming WT and Btk-KO B cells; su-Ag, 20 molec/μm^2^. **(B)** su-Ag central cluster formation frequency in presence of distinct su-Ag densities. **(C)** Values of contact area (estimated by IRM; left) and total su-Ag FL (in AU; right) for IS-forming B cells; su-Ag, 20 molec/μm^2^. **(D)** DIC and IRM images of representative B cells at the time of maximum cell spreading during IS formation. Right, maximum spreading area values (estimated by IRM). **(E)** Fluo4FF-labeled B cells were monitored for Ca^2+^ influx; total Fluo4FF FL values (in AU) over time are shown; data shown as mean ± SD of 20 B cells/condition. Inner bars-graph, AUC values per B cell and per condition; each dot is a cell. **(F)** DIC and fluorescence images of vinculin (green), F-actin (red) and su-Ag (white) for representative IS-forming B cells fixed at 10 min; su-Ag, 20 molec/μm^2^. Right, values of total vinculin and F-actin FL at the IS. **(G)** DIC, IRM and fluorescence su-Ag images at the contact plane of representative IS-forming WT and Btk-KO B cells, untreated or treated with the indicated inhibitors at the specified dose; su-Ag, 20 molec/μm^2^. **(H)** Values of contact area and total su-Ag FL for IS-forming B cells in each condition. **(I)** Total Fluo4FF FL values (in AU) over time are shown for WT (left panel) and Btk-KO (mid- and right panels) B cells in each specified condition; data shown as mean ± SD of 20 B cells/condition. Bar, 2 μm. Each dot in **(B)** represents a single image field, and in **(C–D,F)** and **(H)**, a single cell. Data from a representative experiment are shown in **(B–G)** (*n* = 3); data from representative experiments are shown in I (*n* = 2). ^*^p < 0.05; ^***^p < 0.001; ^****^p < 0.0001 by Student's *t*-test with WT or Btk-KO in each case.

We evaluated IS formation in primary B cells isolated from Btk-knockout (Btk-KO) mice. Their IgM/IgD phenotype was similar to the one for Xid B cells (Supplementary Figure 4C), as reported (21). In presence of distinct su-Ag densities, Btk-KO B cells showed frequencies of su-Ag central cluster formation and values of contact area and total su-Ag aggregation similar to those for WT (Figures 6A–C). Quantification of the maximum spreading area during IS formation showed no alterations compared with control B cells (Figure 6D). BCR-triggered Ca^2+^ influx was nonetheless reduced in Btk-KO B cells, to an extent similar to Xid B cells (Figure 6E). Related to pSMAC molecular assembly, Btk-KO B cells formed the vinculin/F-actin-enriched ring-shaped structure as in WT cells; vinculin recruitment and F-actin levels at the IS were as in controls (Figure 6F). B cells that lack Btk protein thus have no marked alterations in IS formation, in contrast to Xid and Ibru-B cells. Besides, these data also confirmed that the IgM^hi^ (immature) profile of Xid B cells, characteristic also of Btk-KO B cells, is not causing the alteration in synapse formation found in Xid B cells.

We investigated if other Tec family member might assume the Btk functions related to IS formation in B cells. Tec is expressed in B cells and it is activated after BCR crosslinking (30, 31). Tec is able to associate with PIP5K and to activate the PLCγ2/Ca^2+^ axis (4, 32). We analyzed Tec protein expression in B cell lysates from WT (*n* = 4) and Btk-KO (*n* = 7) mice; Tec was increased in Btk-KO B cells compared to controls (Supplementary Figure 4D). We studied inhibitor treatment effects in Btk-KO B cell IS formation. Tec is a potential off-target of ibrutinib (IC_50_ 78 nM) (24); acalabrutinib seems to have minimal effect on Tec kinase activity (29). We treated Btk-KO B cells with 50 nM Ibru or 500 nM Acala, and then assessed them for IS formation using the su-Ag-containing artificial membranes. Inhibitor-treated Btk-KO B cells had reduced contact areas and total su-Ag aggregation at the synapse, as previously observed for inhibitor-treated WT B cells (Figures 6G,H). Calcium influx during IS formation was inhibited by Ibru or Acala in Btk-KO B cells (Figure 6I). Studies on F-actin at the synapse by IF showed no defects in inhibitor-treated compared to untreated Btk-KO B cells (Supplementary Figures 4E,F). Data suggested that 500 nM Acala affected Tec kinase activity in Btk-KO B cells. To impair Btk kinase and minimize the effect on Tec, we treated WT and Btk-KO B cells with 10 nM Acala, a dose ten-times below the IC_50_ value for Tec (93 nM) but in the range of the IC_50_ for Btk (5 nM) (29). Ten nanomolar-Acala-treated WT B cells showed significantly reduced values of contact area and su-Ag aggregation at the IS, as well as decreased calcium influx; su-Ag aggregation was unaffected while contact area and calcium influx were reduced in Btk-KO B cells (Figures 6G–I, Supplementary Figure 4G). Data suggested that, in Btk-KO B cells, Tec adopts the Btk functions needed for IS formation.

### Btk-KO B cells have impaired MTOC polarization while normal cell activation and proliferation

**Figure 7:**
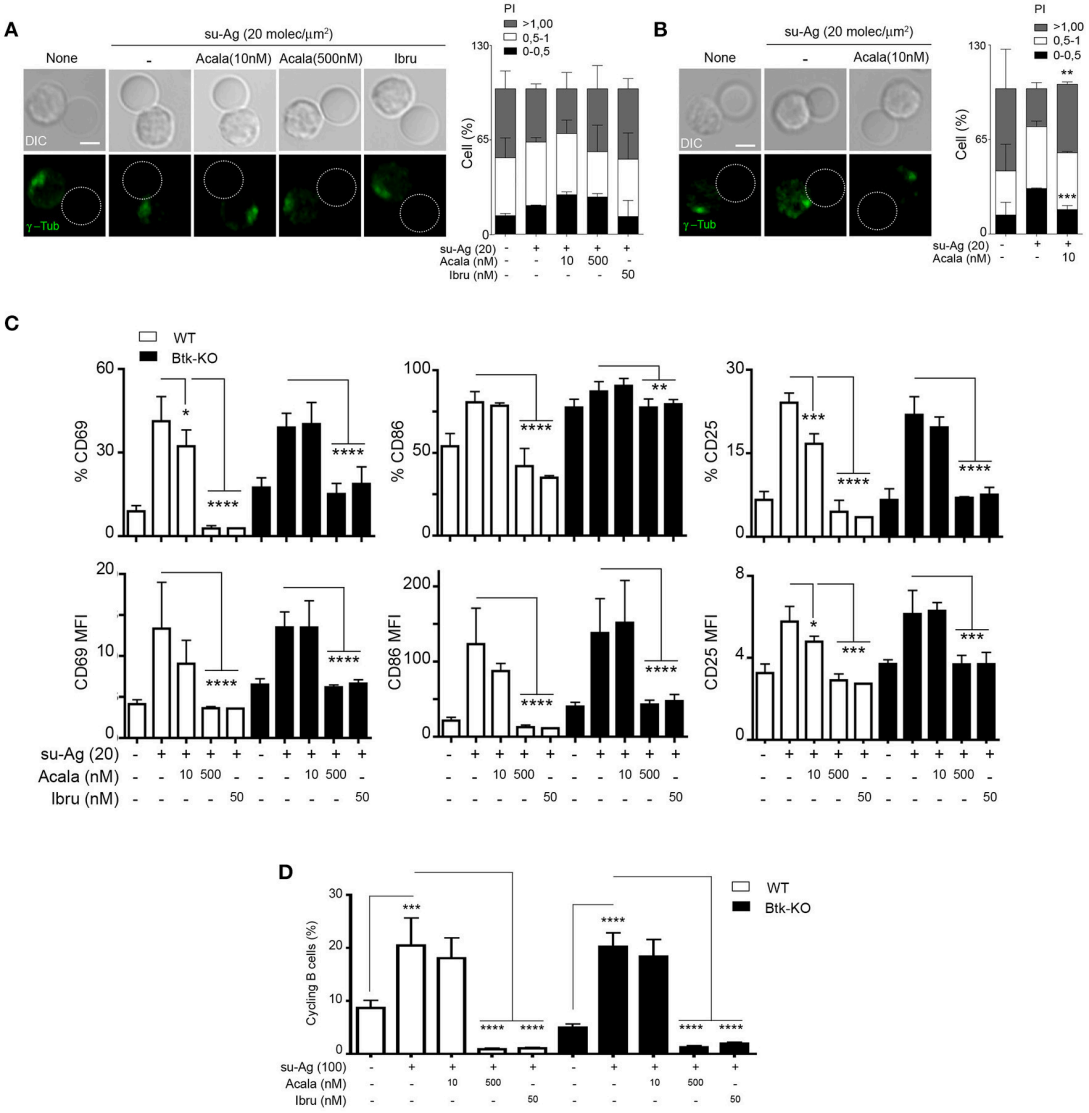
Btk-KO B cells have impaired MTOC polarization, but no significant alterations in cell activation or proliferation**. (A)** DIC and fluorescence γ-tubulin (γ-tub; green) images are shown of representative Btk-KO B cell/pseudo-APC conjugates, fixed at 30 min, for each indicated condition. Dashed circle, pseudo-APC; right, frequency of B cells as in A, in the specified polarity index (PI) groups; data shown as mean ± SD of 60 B cells in each case. **(B)** As in **(A)** but for WT B cells in the specified conditions. **(C)** WT and Btk-KO B cells, untreated or treated with the indicated inhibitors, were co-cultured with pseudo-APC, unloaded (none) or su-Ag-loaded (20 molec/μm^2^; su-Ag-20) at 1:1 ratio, 20 h. Frequencies of B cells expressing CD69, CD25 or CD86 (top) and MFI values for these markers (bottom) in each condition and for each B cell type are shown. **(D)** Violet-labeled WT and Btk-KO B cells were co-cultured with pseudo-APC, unloaded (none) or su-Ag-loaded (100 molec/μm^2^, su-Ag-100, ratio 1:1), with IL-4, 72 h. Frequencies of cycling B cells (with diluted violet-tracer level) in each condition and for each B cell type are shown. Data in **(A)** are the merge of three experiments (*n* = 3), and in **(B)** of two experiments (*n* = 2); data in **(C,D)** are the mean ± SD of three experiments (*n* = 3). ^*^p < 0.05; ^**^p < 0.01; ^***^p < 0.001; ^****^p < 0.0001 by Student's *t*-test with WT or Btk-KO in presence of su-Ag in each case. Bar 2 μm.

We evaluated Btk-KO B cell ability to recruit the MTOC to the IS formed with su-Ag-loaded pseudo-APC. At the time analyzed (30 min), Btk-KO B cells did not significantly polarize the MTOC to the IS compared to the condition in absence of su-Ag or to su-Ag exposed WT B cells (Figures 7A,B). Inhibitor-treated Btk-KO B cells showed no changes (Figure 7A); 10 nM-Acala decreased MTOC polarization in WT B cells (Figure 7B).

We studied later activation events in Btk-KO compared to WT B cells; for that, we did co-cultures of B cells with unloaded or su-Ag-loaded psedo-APC at 1:1 ratio and analyzed CD69, CD86 and CD25 surface expression at 20 h. In these experimental conditions, we detected no significant differences in the frequency and expression levels of the three markers between Btk-KO B cells and controls exposed to su-Ag (Figure 7C). Ibru- or 500 nM-Acala-treatment of Btk-KO B cells avoided activation markers upregulation (Figure 7C). Ten nanomolar-Acala-treated Btk-KO B cells behaved as untreated ones, while WT showed reduced levels for CD69 and CD25 compared to untreated (Figure 7C). To assess proliferation, we performed co-cultures of violet-tracer-labeled B cells with pseudo-APC, in absence or presence of su-Ag, at ratio 1:1 for 72 h; when indicated, B cells were treated with the Btk inhibitors 1 h before adding the pseudo-APC to the culture. Cycling cells frequencies for Btk-KO B cells were comparable to those for WT; Ibru and 500 nM-Acala treatment inhibited proliferation in both B cell types. We observed no significant defects in proliferation for WT or Btk-KO B cells treated with 10 nM-Acala (Figure 7D). Then, in the experimental approach used, Tec seemed to control activation markers expression and cell proliferation in absence of Btk.

## Discussion

Here we report distinct roles for Btk in B cell IS formation. PH domain-mediated Btk recruitment to the plasma membrane is required for the B cell capacity to trigger IS formation and for appropriate pSMAC assembly; the shuttling/scaffold activities and the regulation of PIP_2_ production at the antigen recognition site appeared to be involved. Btk-kinase function drives the quantity of antigen gathered at the cSMAC by controlling the PLCγ2/Ca^2+^ axis. Impaired membrane recruitment or kinase activity of Btk greatly affected MTOC polarization, B cell activation and proliferation. These data also indicate the equal relevance of IS architecture and of quantity of antigen accumulated at the synapse to trigger B cells.

*Via* PIP5KI transport to the plasma membrane, Btk regulates PIP_2_ production and provides substrate for second messenger generation by PI3K and PLCγ2. Both PI3K and PLCγ2 activation are important for B cell IS formation (6, 13). Nonetheless, Btk-kinase inhibition led to larger defects in PI3K and PLCγ2/Ca^2+^ activities than Xid-Btk, and did not interfere with the B cell capacity to elicit IS formation and to assemble the pSMAC. Other mechanisms might thus be altered in Xid B cells. PIP_2_ regulates the actin cytoskeleton and adhesion site dynamics, acting as a scaffold for proteins such as vinculin, talin, and WASP; a local increase in PIP_2_ drives actin polymerization (27, 33). We propose that PIP_2_ generation at the B cell contact site with the APC drives WASP recruitment, which associates and activates Arp2/3 actin nucleator to trigger actin polymerization; B cells lacking WASP have altered pSMAC formation (15). PIP_2_-mediated talin and vinculin recruitment aid in adhesion-site assembly (LFA-1/ICAM-1 domain). Vinculin stabilizes the pSMAC by linking integrin/talin complexes to the F-actin-rich ring through its own binding to talin and F-actin; vinculin loss at the mature synapse leads to pSMAC destabilization, allowing B cells to move carrying the antigen cluster at the trailing edge (12). We detected reduced PIP_2_ production in Xid B cells, what correlated with lower recruitment of WASP, talin and vinculin. Diminished WASP implies less Arp2/3 and thus decreases actin polymerization, as detected by the reduced F-actin levels at the Xid B cell IS. In addition, lack of pSMAC organization might be due to impaired vinculin recruitment. Several reports showed the importance of actin cytoskeleton and receptor organization at the plasma membrane to govern BCR signaling (16, 34–36). Although additional studies remain, we propose that the Xid mutation in Btk disrupts actin network and adhesion site dynamics, both responsible for B cell inability to respond to antigen.

Btk-kinase inhibition significantly reduced B cell spreading, resulting in less su-Ag accumulation at the cSMAC; those events related with decreased PLCγ2 activation and Ca^2+^ influx. These results coincide with the role for the PLCγ2/Ca^2+^ axis in BCR microclusters assembly and cell spreading (6, 26). Rap GTPases are also involved in those events through cofilin activation (35). Ibru-B cells however showed strong cofilin activation at the spreading phase, suggesting that Btk-kinase regulates cell spreading and antigen gathering at the IS by cofilin-independent mechanisms. In planar artificial membranes bearing only antigen, Xid B cells have defects in cell spreading and BCR microcluster formation (8). In our model, those Xid B cells able to form the IS showed no alterations in cell spreading or antigen accumulation. The ICAM-1/CXCL13 presence might account for the difference in results, as LFA-1/ICAM-1 aids antigen gathering, and CXCL13/CXCR5 promotes LFA-1 activation and actin rearrangement (9, 11).

BCR-triggered integrin activation involves the Btk/PLCγ2 axis in DT40 B cells (5). In accordance, Btk kinase-inhibited primary B cells showed reduced LFA-1 activation (less cell spreading) as well as nearly undetectable Ca^2+^ influx after su-Ag encounter. Those B cells formed a pSMAC, which suggests no major defects in active LFA-1 clustering at the IS. Btk shuttling/scaffold activity, in contrast, seems dispensable for BCR-triggered LFA-1 activation (normal cell spreading) but critical for integrin clustering (pSMAC assembly). The limited vinculin, talin and WASP recruitment at the Xid B cell IS stresses the defects in adhesion-site assembly at the plasma membrane.

B cell maturation is affected in Xid and Btk-KO mice; splenic B cells display high expression of surface IgM, characteristic of immature populations (transitional stages T1/T2). Transitional B cells are more sensitive to BCR stimulation than mature B cells (37). A fraction of T1/T2 B cells does not centralize the antigen in a single central cluster at the synapse (38). Our data showed that IgM^hi^ B cells accumulate F-actin at the IS and form F-actin rings. The latter together with the normal IS formation found in Btk-KO B cells, suggested that Xid B cell synapse defects are not related to their immature stage but to Btk dysfunction. Nonetheless, more studies are needed comparing Xid B cells with the equivalent WT immature populations in relation to IS formation and BCR signaling. Related to that, the lack of cofilin phosphorylation might be due to Btk dysfunction or the immature stage of Xid B cells.

The absence of synapse defects in B cells lacking Btk indicated that other Tec family member might assume those Btk functions. The increased Tec protein levels and the inhibitors effect on Btk-KO B cells pointed to Tec activity as the compensatory mechanism. Btk mutant proteins, as Xid Btk, could also act as a dominant negative impairing not only Btk but also Tec activities, since they share some relevant interaction partners. Previous studies reported impaired BCR-triggered activation and proliferation for Btk-KO B cells when stimulated *in vitro* [reviewed in (1)]. In our studies, Btk-KO B cells showed activation and proliferation responses similar to WT. These different results might be due to the experimental approach used.

Both Xid and Ibru-B cells had great defects in MTOC polarization to the IS, needed for membrane trafficking and antigen extraction/processing. Studies with soluble antigen stimulation showed reduced BCR internalization and antigen presentation in Xid and Btk-kinase inhibitor-treated B cells (7). Cell activation and proliferation were similarly compromised in Xid and Ibru-B cells. Btk functional deficiency in shuttling/scaffold and/or kinase activities hence causes severe defects in the antigen-triggered B cell response, either by affecting the ability to form the IS and rightly assemble the pSMAC or by altering the PLCγ2/Ca^2+^ axis and antigen gathering. We propose a model that integrates the distinct Btk roles in B cell IS formation (Supplementary Figure 5). The Xid mutation impairs Btk recruitment to the membrane, altering IS formation ability and assembly; inhibiting Btk kinase activity, BCR signaling is diminished (PLCγ2/Ca^2+^ and downstream targets) while the IS architecture is normal. In absence of Btk protein, Tec assume these Btk functions downstream of the BCR. Besides, Xid-Btk or just the inhibition of the kinase activity might exert dominant negative effects, by capturing proteins (as PIP5KI) or blocking protein interaction sites (SH2-binding sites), compared to complete lack of Btk. Our data also underline the importance of pSMAC assembly and correct IS architecture for B cell activation, which are as important as the quantity of antigen detected by the BCR. Impaired IS molecular assembly might relate with BCR signaling defects due to changes in signaling molecules recruitment/location, leading to B cell activation failure.

## Author contributions

SR-G designed parts of the study, performed experiments, analyzed the data and assisted in manuscript preparation; SM-C and SG performed some experiments, analyzed the data and assisted in manuscript preparation. MdB and RH provided Btk-KO mice and input into the project; YC designed and supervised all aspects of the work and wrote the manuscript.

### Conflict of interest statement

The authors declare that the research was conducted in the absence of any commercial or financial relationships that could be construed as a potential conflict of interest.
