# Supplementary material for: Distinct Roles for Bruton's Tyrosine Kinase in B Cell Immune Synapse Formation
Source: Front Immunol. 2018 Sep 6;9:2027. doi: 10.3389/fimmu.2018.02027 (PMC6136277; doi:10.3389/fimmu.2018.02027)
Supplement: Supplementary file 1 [file Image_1.PDF]

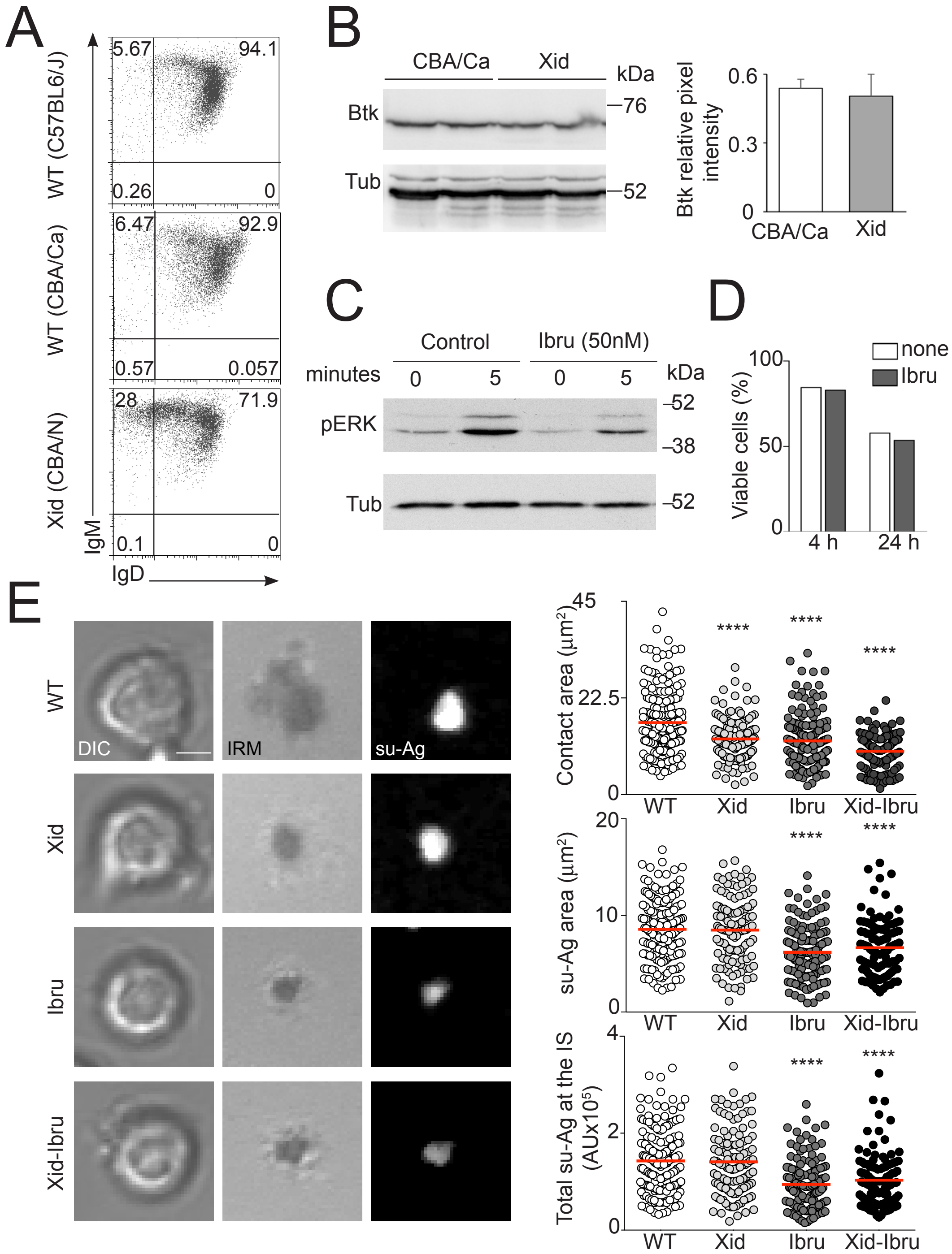

Supplementary Figure 1. Primary B cell phenotype and ibrutinib treatment. (A) Representative dot plots of surface IgM/IgD expression in freshly isolated spleen B cells from WT C57BL/6J and CBA/Ca mice, and from Xid (CBA/N) mice. (B) Immunoblot of freshly isolated WT (CBA/Ca) and Xid (CBA/N) B cell lysates, probed with specific antibodies. Quantification of Btk intensity normalized to Tubulin (loading control) by densitometry is shown; data are the mean ± SD of two WT or Xid B cell lysates. (C) Immunoblot of untreated or ibrutinib-treated (Ibru, 50 nM) WT B cells stimulated in Ab-coated plates, probed with antibodies to phosphorylated ERK1/2 (p-ERK1/2, Y202/Y204) and to tubulin (Tub; loading control). (D) Frequency of viable B cells cultured alone (none) or with Ibru (50 nM) over time, estimated by flow cytometry (using cell size/FSC versus complexity/SSC dot plots). (E) DIC, IRM and fluorescence su-Ag images at the contact plane of representative IS-forming WT and Xid B cells, untreated or Ibru-treated; antigen, 20 molec/μm^2. Bar, 2 μm. Values of cell contact area (estimated by IRM), su-Ag aggregate area (cSMAC) and total su-Ag fluorescence (FL) (in arbitrary FL units, AU) for each B cell type are shown; right. Each dot represents a single cell. Data of a representative experiment (n = 3). ****, p<0.0001 by Student's t-test with WT in each case.
