# Supplementary figures and images for: Distinct Roles for Bruton's Tyrosine Kinase in B Cell Immune Synapse Formation
Source: Front Immunol. 2018 Sep 6;9:2027. doi: 10.3389/fimmu.2018.02027 (PMC6136277; doi:10.3389/fimmu.2018.02027)

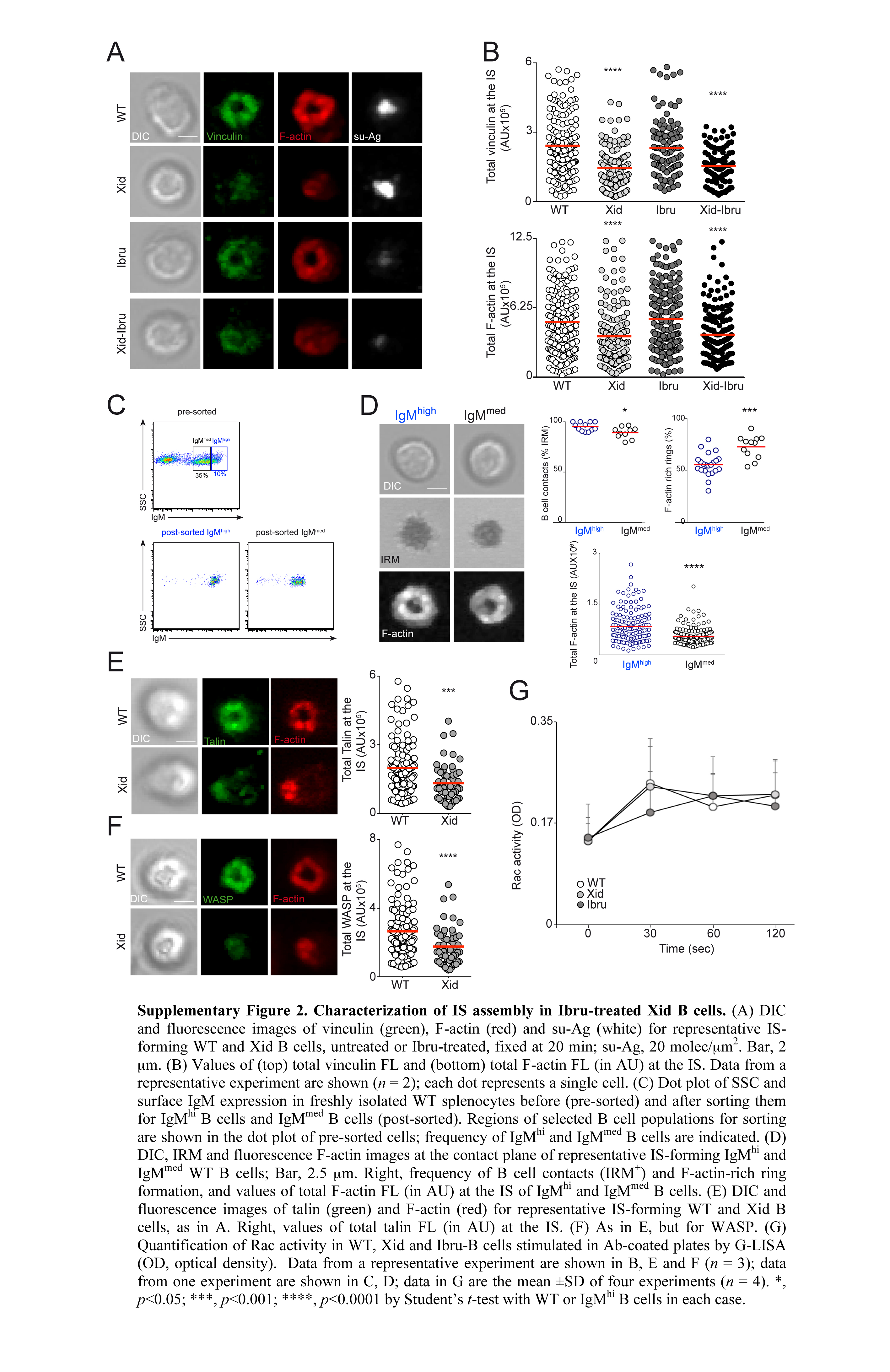

Supplement: Supplementary file 2 [file Image_2.JPEG]

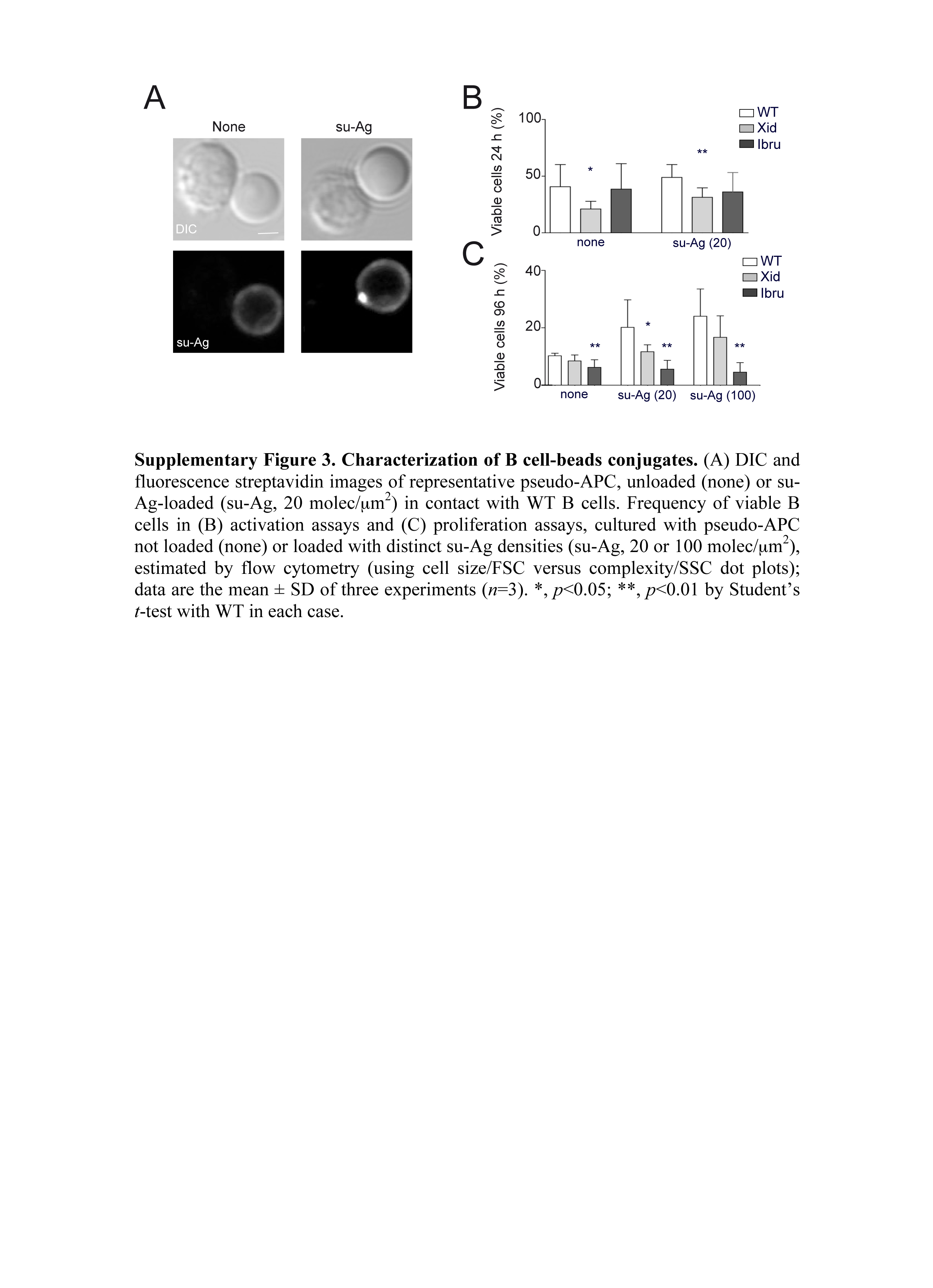

Supplement: Supplementary file 3 [file Image_3.JPEG]

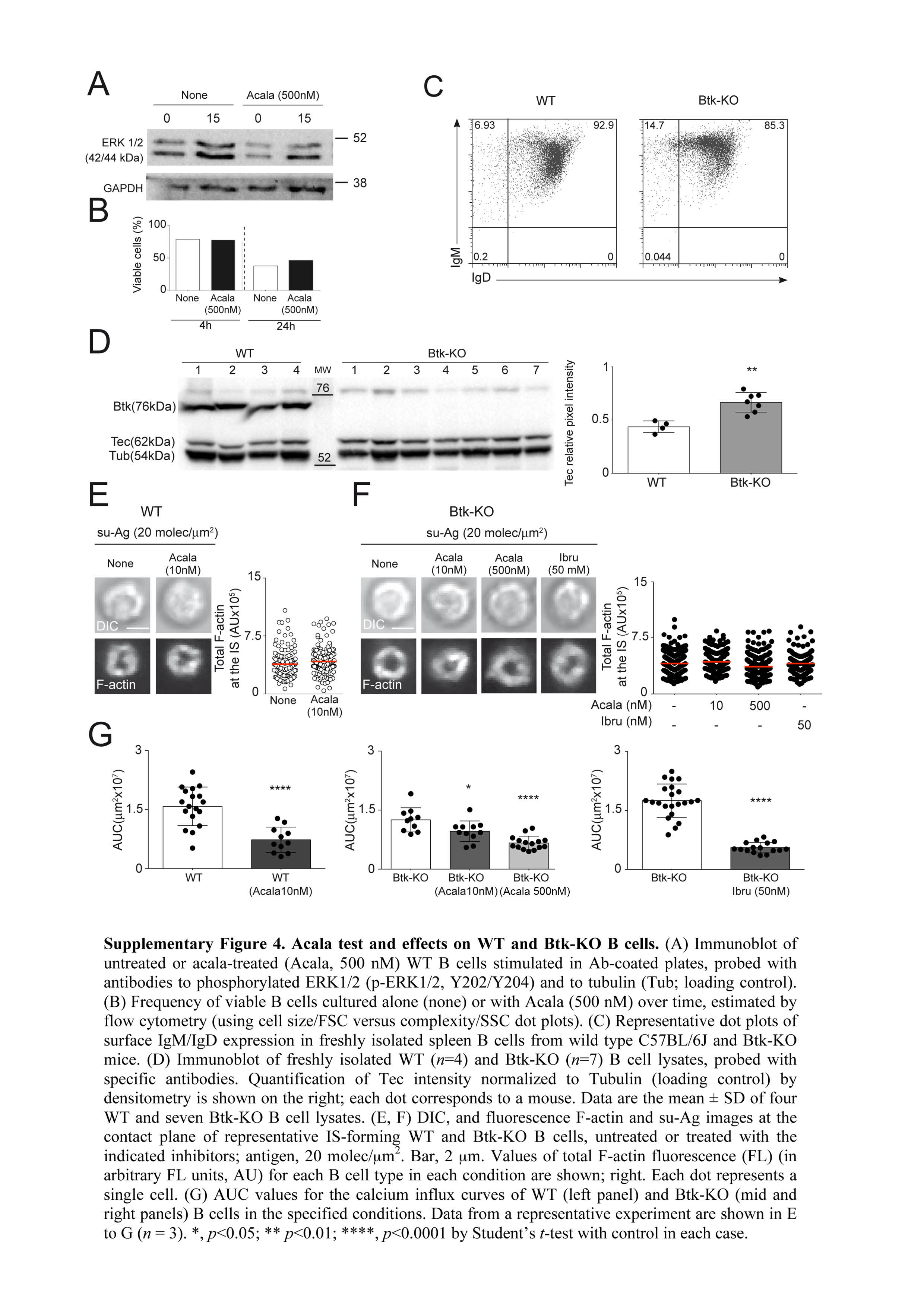

Supplement: Supplementary file 4 [file Image_4.JPEG]

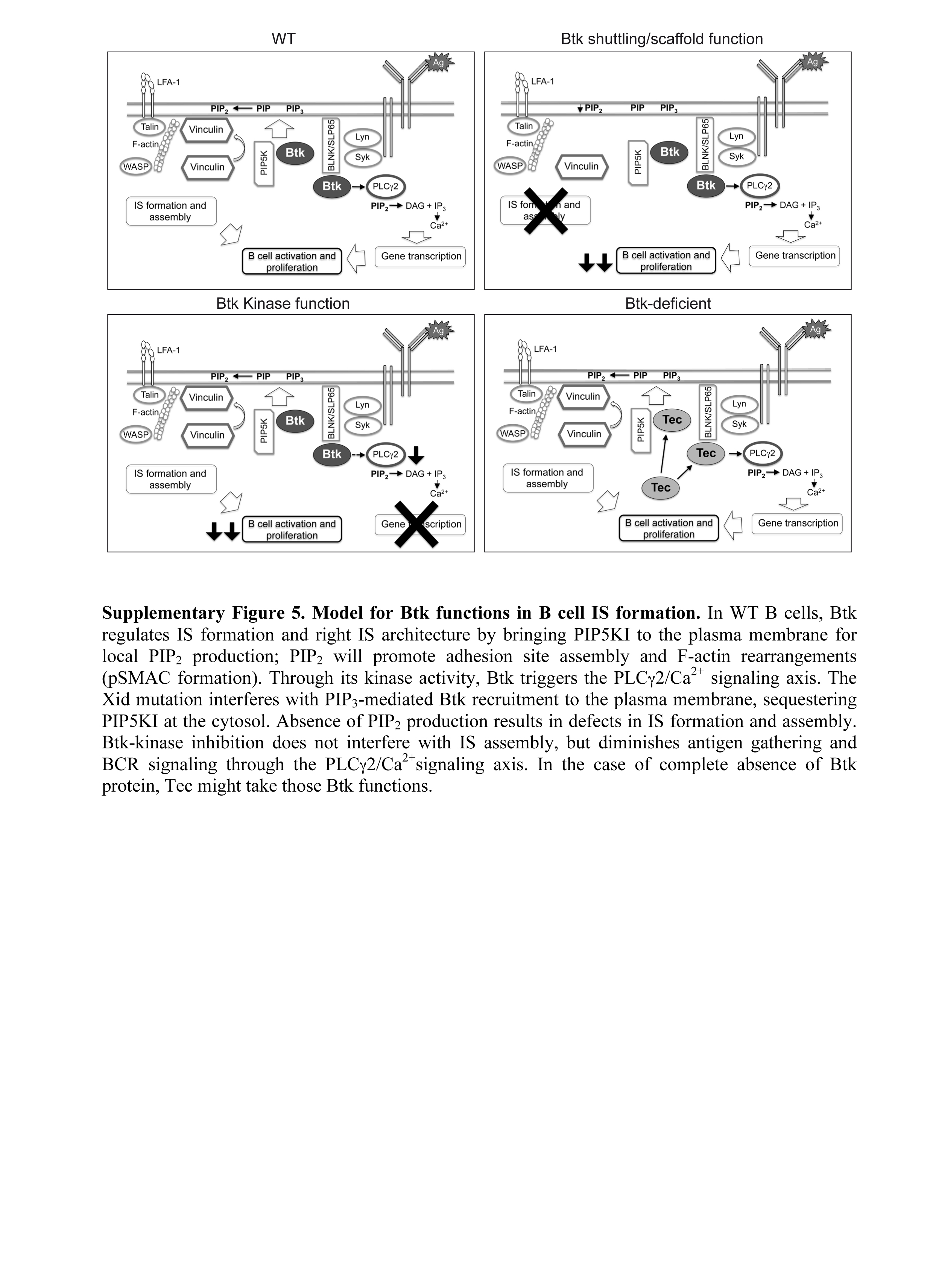

Supplement: Supplementary file 5 [file Image_5.JPEG]
